# RNA sequencing analysis reveals the competing endogenous RNAs interplay in resected hepatocellular carcinoma patients who received interferon-alpha therapy

**DOI:** 10.1186/s12935-021-02170-w

**Published:** 2021-09-06

**Authors:** Yibin Wu, Longrong Wang, Xiaoshuang Wang, Yiming Zhao, Anrong Mao, Ning Zhang, Jiamin Zhou, Qi Pan, Weiping Zhu, Lu Wang

**Affiliations:** Department of Hepatic Surgery, Fudan University Shanghai Cancer Center, Shanghai Medical College, Fudan University, Shanghai, 200032 PR China

**Keywords:** Hepatocellular carcinoma, Interferon-alpha, RNA sequencing, Competing endogenous RNAs, MARCH3, Tumor-infiltrating

## Abstract

**Background:**

Interferon-alpha (IFN-α) is a general therapeutic regimen to be utilized in hepatocellular carcinoma (HCC). However, regulatory mechanisms of IFN-α on competing endogenous RNAs (ceRNAs) level in anti-HCC relapse are rarely understood.

**Methods:**

HCC patients with and without IFN-α treatment were calculated to analyze the expression profile of mRNA, long non-coding RNA (lncRNA), microRNA (miRNA), and circular RNA (circRNA) by RNA sequence, and significant differential expression (DE) of these types of RNAs were selected for further analysis. A ceRNA regulatory network was constructed to explore the potential mechanisms of IFN-α intervention on anti-HCC relapse. Finally, the potential prognostic associated genes among these DE RNAs were identified.

**Results:**

Totally, 556 mRNAs, 120 circRNAs, 87 lncRNAs, and 96 miRNAs were differentially expressed in patients who received IFN-α treatment. A ceRNA regulatory network including a circRNA-miRNA-mRNA network which composed of 4 up- and 10 down-regulated circRNAs, 8 up- and 5 down-regulated miRNAs, 28 up- and 9 down-regulated mRNAs, and a lncRNA-miRNA-mRNA network which composed of 10 up- and 3 down-regulated lncRNAs, 11 up- and 5 down-regulated miRNAs, 28 up- and 10 down-regulated mRNAs was constructed. Gene enrichment and pathway analysis revealed that the ceRNA network was associated with immune-related pathway and corresponding molecular function in patients who accepted IFN-α treatment. Next, we identified 3 most relevant to IFN-α treatment to HCC among these DE RNAs, namely FAM20A, IGFBP4 and MARCH3, as the prognostic associated genes for HCC. Furthermore, MARCH3 expression correlated with infiltrating levels of tumor infiltrating immune cells (TICCs) in HCC. MARCH3 expression also showed strong correlations with the gene markers of diverse immune cells in HCC.

**Conclusion:**

Our data discovered a novel ceRNA network in HCC patients receiving IFN-α therapy, which might lay the foundation for better understand the regulatory mechanism of IFN-α treatment.

## Background

Hepatocellular carcinoma, one of the most cancer related death malignancies, is highly prevalent in China [1]. Despite surgery as the most effective method for HCC patients in early stage, patients often suffer with high recurrence with the rate up to 50% in the 3 years [2–4]. Thus, adjuvant therapy is increasing attractive and becomes the research focus. IFN-α is an antiviral cytokine which has anti-proliferation and immunomodulatory properties against malignant tumors [5], and has been reported that it could inhibit tumor metastasis in HCC xenografts model [6], and in our clinical trials [7]. However, the characteristics of patients who can benefit from IFN-α are not consistent.

Yang el al. have reported that the increased expression of IFIT3 in tumor tissue can be regarded as an indicator for predicting the response of IFN-α therapy [8]. Retinoic acid-inducible gene-I (RIG-I), another interferon-stimulated gene, has also been proven as a predictive indicator. Namely, patients with low RIG-I expression always presented shorter survival and to poorer response IFN-α therapy [9]. In addition, we found that dihydropyridine dehydrogenase (DPYD), a pyrimidine catabolic enzyme, was downregulated in HCC tissues depending on the dose of IFN-α, and might be a potential prognostic biomarker and a therapeutic target for HCC [10]. Although many researches have stratified the potential patients who might benefit from IFN-α therapy, no agreement has been reached.

Competing endogenous RNA (ceRNA) network is an entrant which can indirectly regulate mRNAs via competitively binding miRNAs. And in this theory, ceRNA refers to all the transcripts which can be used as the targets of miRNAs, namely including circRNA, pseudogene RNA, and lncRNA [11]. The mechanisms of ceRNA network regulating IFN-α mRNA level have been widely reported. Specifically, Tominori et al. have reported ceRNAs, including IFN-α1 antisense RNA (AS) and cellular mRNAs, that bind to miR-1270 could precisely maintain the level of the type-I IFN and function on the innate immune system [12]. In vitro experiments also have indicated that several lncRNA were differentially expressed to induce the IFN pathway under the IFN-stimulation [13]. In addition, ceRNA has also been researched in the area of liver cancer. Analyses of the data from The Cancer Genome Atlas (TCGA) database, Zhao et al. has discovered a HCC-related ceRNA network which can be used as the prognostic factor to reveal cancer progression [14]. Another larger-clinical-sample research further indicated that the ceRNA network might aid to elucidate the mechanism of HCC pathogenesis [15]. However, limited studies have reported to reveal the mechanisms on ceRNA regulating the IFN-associated pathways in HCC based on the sequencing of clinical samples.

Currently, utilizing the clinical samples, we try to probe the possible mechanism on how the IFN-α functions on cancer cells based on RNA sequencing. TIMER and GEPIA databases were used to further analyze the correction between MARCH3 expression and tumor-infiltrating in HCC. The results may lay the foundation on better comprehending the functional process of IFN-α treatment on HCC, and stratified potentially beneficial patients who received the IFN-α therapy.

## Materials and methods

### Patients

Totally, 9 patients who underwent radical surgery at Fudan University Shanghai Cancer Center (Shanghai, China) were enrolled in this study. Among these patients, peripheral blood samples (PBMCs) were taken from each patients two weeks after surgery (Regarded as group A). Next, another 9 PBMCs were further collected from these patients after injected with interferon-alpha (30 μg twice a week; recombinant human interferon a-2b; Shenzhen Kexing Bioengineering Co., Ltd.) after two weeks (Regard as group B). The clinicopathological characteristics of these patients are shown in Table 1. Prior patient consent was obtained, and the study was approved by the Ethics Committee of Fudan University Shanghai Cancer Center.

**Table 1:** The clinicopathological characteristics in 9 HCC patients

Characteristics	Number of the population
Age (years)	
< 50	3
≥ 50	6
Gender	
Male	7
Female	2
HBV infection	
Yes	9
No	0
Advanced fibrosis or cirrhosis	
Yes	5
No	4
Child–Pugh scores	
A	9
B	0
C	0
AJCC stage	
T1	7
T2	2
T3	0
T4	0
Tumor morphology	
Nodular	9
Blocky pattern	0
Neoadjuvant therapy	
Yes	0
No	9
Tumor size (cm)	
< 5	9
≥ 5	0
Grade	
Well differentiated	3
Moderately differentiated	6
Poorly differentiated	0
Surgical margin	
Positive	0
Negative	9

*HCC* hepatocellular carcinoma, *AJCC* American Joint Committee on cancer

### Isolation of peripheral blood mononuclear cells and sequencing

Peripheral blood mononuclear cells (PBMCs) were respectively isolated from the peripheral blood of all the participants based on the previous method with some modifications [16]. The blood samples (3 ml) were collected form patients. Firstly, the peripheral blood was layered and centrifuged (950 g, 30 min). Then the Ficoll-Histopaque layer was collected and stored for the following experiments. The trypan blue exclusion test was utilized to assay the cell viability. Total RNA was isolated from the extracted PBMC by TRIzol reagent (Invitrogen, USA) following the manufacturer’s protocol. With the aid of 2100 Bioanalyzer (Agilent, USA) and NanoDrop Spectrophotometer (NanoDrop, USA), the RNA quality was detected. The A260/280 ratio of the 8 samples were ranged from 1.8 to 2.0.

### RNA library construction and RNA sequencing

Total RNA was used for library preparation using TruSeq Stranded Total RNA with Ribo-Zero Gold (Illumina, USA). rRNA was removed and strand-specific RNA-seq libraries were prepared following the manufacturer’s instructions. Briefly, ribosome depleted RNA was fragmented and then used for first- and second-strand complementary DNA (cDNA) synthesis with random hexamer primers. dUTP mix was used for second-strand cDNA synthesis, which allows for the removal of the second strand. The cDNA fragments were end repaired, A-tailed and ligated with indexed adapters. The ligated cDNA products were purified and treated with uracil DNA glycosylase to remove the second-strand cDNA. Purified first-strand cDNA was subjected to PCR amplification, and the libraries were quality controlled with a Bioanalyzer 2200 (Agilent, Santa Clara, CA) and sequenced by HiSeq Xten (Illumina, San Diego, CA) on a 150 bp paired-end run.

### miRNA library construction and miRNA sequencing

The NEBNext Small RNA Library Prep Set for Illumina was used to generate the miRNA sequencing library. Briefly, the total RNA was ligated using the manufacturer-supplied RNA 3′ and RNA 5′ adapters. And the ligated miRNA was subjected to a RT step for the first strand cDNA synthesis. Then an index PCR was performed to add sample specific index sequence and Illumina sequence adapters. The libraries were gel purified and quality controlled with Bioanalyzer 2200 (Agilent, Santa Clara, CA) and sequenced on HiSeq Xten (Illumina, San Diego, CA) on a 150 bp paired-end run.

### CircRNA identification

Unmapped Reads was collected to identify the circRNA utilizing BWA mem (bwa mem -t 1 -k 16 -T 20): Partial alignments of segments within a single read that mapped to (i) regions on the same chromosome and no more than 1 Mb away from each other (ii) on the same strand (iii) but in reverse order, were retained as candidates supporting head-to-tail junction. The strength of potential splicing sites supported by these candidate head-to-tail junction reads was then estimated using MaxEntScan33. The exact junction site was determined by selecting the donor and acceptor sites with the highest splicing strength score. Candidate circRNAs were reported if the head-to-tail junction was supported by at least two reads and the splicing score was greater than or equal to 10.

### Expression analysis

To estimate the expression of circRNA, we re-aligned all the unmapped reads to the circRNA candidates by using the BWA-mem under following parameter (bwa mem -t 1 -k 16 -T 20). As for most of the circRNAs there is no direct evidence for their exact sequence, we filled in the sequence using existing exon annotation. Sequence at the 5′ end was concatenated to the 3′ end to form circular junctions. Reads that mapped to the junction (with an overhang of at least 6 nt) were counted for each candidate.

### Date processing and differentially expressed RNAs screening

Raw reads were screened with an in-house pipeline which had been internally validated. To acquire the clean reads, adaptor, low quality, and high unknown bases were trimmed. Genome GRCh38 was utilized as the human reference background and the analyzing tool was Bowtie [17] or TopHat [18]. The CIRI software was used to scan for paired chiastic clipping signals (CIRI: an efficient and unbiased algorithm for de novo circular RNA identification). RNA sequences were predicted based on the junction reads and GT-AG cleavage signals. The expression levels of RNAs were counted by using the reads per kilobase transcriptome per million mapped reads (RPM) method. The differentially expressed (DE) RNAs (including miRNAs, mRNAs, or circRNAs) between samples were identified by the DEGseq or Cufflinks package under the R environment. The cut-off criteria were designed as log2 (Fold_change) > 2 with the p value < 0.01. The data was used to further establish a ceRNA regulatory network.

### Establishment of a ceRNA regulatory network

Firstly, the miRWalk (http://mirwalk.umm.uni-heidelberg.de/) and TargetScan (http://www.targetscan.org/mamm_31/) software were respectively utilized to predict the target genes and target circRNAs for the DE miRNAs. The starbased website (http://starbase.sysu.edu.cn/) was utilized to predict the target genes for DE circRNAs. Secondly, the possible functional relationships between DE RNAs were further probed. Significantly DE mRNAs and DE circRNAs were analyzed the Pearson correlation coefficient to determine whether presented co-expressed, and the Pearson correlation coefficient index was designed over than 0.9 with an adjusted p value < 0.1. The whole ceRNA network including the miRNA-circRNA regulatory network, the miRNA-target gene regulatory network, and the circRNA-mRNA co-expression network were constructed. A ceRNA regulatory network was constructed to visualize the interaction between the selected miRNAs and mRNAs or IncRNAs related with the effects of IFN-α on HCC patients by Cystoscope (version 3.7.2).

### Gene set enrichment analysis

Then Gene Ontology (GO) enrichment and Kyoto Encyclopedia of Genes and Genomes (KEGG) pathway were analyzed on these DE RNAs, including DE mRNAs and the target genes of DE miRNAs and DE circRNAs by using Gene Set Enrichment Analysis (GSEA). Merely when P value < 0.05, the analysis results were regarded as owning the statistical significance.

### Survival analysis

Kaplan–Meier Plotter (KM Plotter) (http://kmplot.com/analysis/) is an online tool to assess the effect of 54 k genes (mRNA, miRNA and protein) on survival in 21 cancer types including liver, breast, lung and gastric cancer. We used KM plotter database to reveal the survival data of these genes including FAM20A, IGFBP4 and MARCH3 on hepatitis B virus (HBV) related HCC.

### TISIDB database analysis

The TISIDB (http://cis.hku.hk/TISIDB) is an integrated repository portal for tumor-immune system interactions. The database includes genomics, transcriptomics and clinical data of 30 cancer types from TCGA. We used the data from TISIDB to reveal the immunological status of MARCH3 in HCC.

### Single cell sequence analysis

Tumor Immune Single Cell Hub (TISCH, http://tisch.comp-genomics.org) is a comprehensive web portal that interacting single-cell transcriptome visualization for tumor microenvironment (TME). In this study, we used TISCH to analyze the correlation of MARCH3 expression with TIICs in HCC. The data was process to be visualized as several clusters by using t-distributed Stochastic Neighbor Embedding (t-SNE) though the Seurat R package.

### TIMER database analysis

TIMER is a comprehensive resource for systematic analysis of immune infiltrates across diverse cancer types (https://cistrome.shinyapps.io/timer/). It includes 10,897 samples across 32 cancer types from TCGA to estimate the abundance of immune infiltrates. We used TIMER to investigate the correlations between MARCH3 expression with the abundance of immune infiltrates, including B cells, CD4 + T cells, CD8 + T cells, neutrophils, macrophages, and dendritic cells, via gene modules. In addition, we also analyzed the correlations between MARCH3 expression and gene markers of the different tumor infiltrating immune cells. The relative gene markers are ever reported in literature.

### Gene correlation analysis in GEPIA

GEPIA (http://gepia.cancer-pku.cn/) is an online tool for analyzing the RNA sequencing expression data of 9736 tumors and 8587 normal samples from the Cancer Genome Atlas and the Genotype Tissue Expression (GTEx) projects. We further explored the expression of the represented genes and confirmed the correlation of MARCH3 with T cell exhaustion via analyzing GEPIA dataset. The Spearman method was used to determine the correlation coefficient.

### Statistical analysis

Baseline characteristics of the two groups were compared using the Chi square test or Fisher’s exact test. Survival curves were depicted by the “Survival” package under the R environment with the Kaplan–Meier method. The survival data originated from the cBio Cancer Genomics Portal (cBioPortal) (https://www.cbioportal.org/). The p value < 0.05 was regarded as the cut-off threshold for significant meaning. All statistical analyses were performed using SPSS 20.0 software (IBM, USA).

## Results

### Basic characteristics of the study population

In this study, 9 patients diagnosed as HCC who accepted IFN-α treatment after surgical resection were included. Among the HBV related HCC patients, 7 patients were male while 2 were female. All of the patients were diagnosed as diagnosed as early HCC with the normal liver function. No patients accepted neoadjuvant therapy, and all of them had negative surgical margin (Table 1).

### Differentially expressed mRNA, miRNA and circRNA

**Fig. 1:**
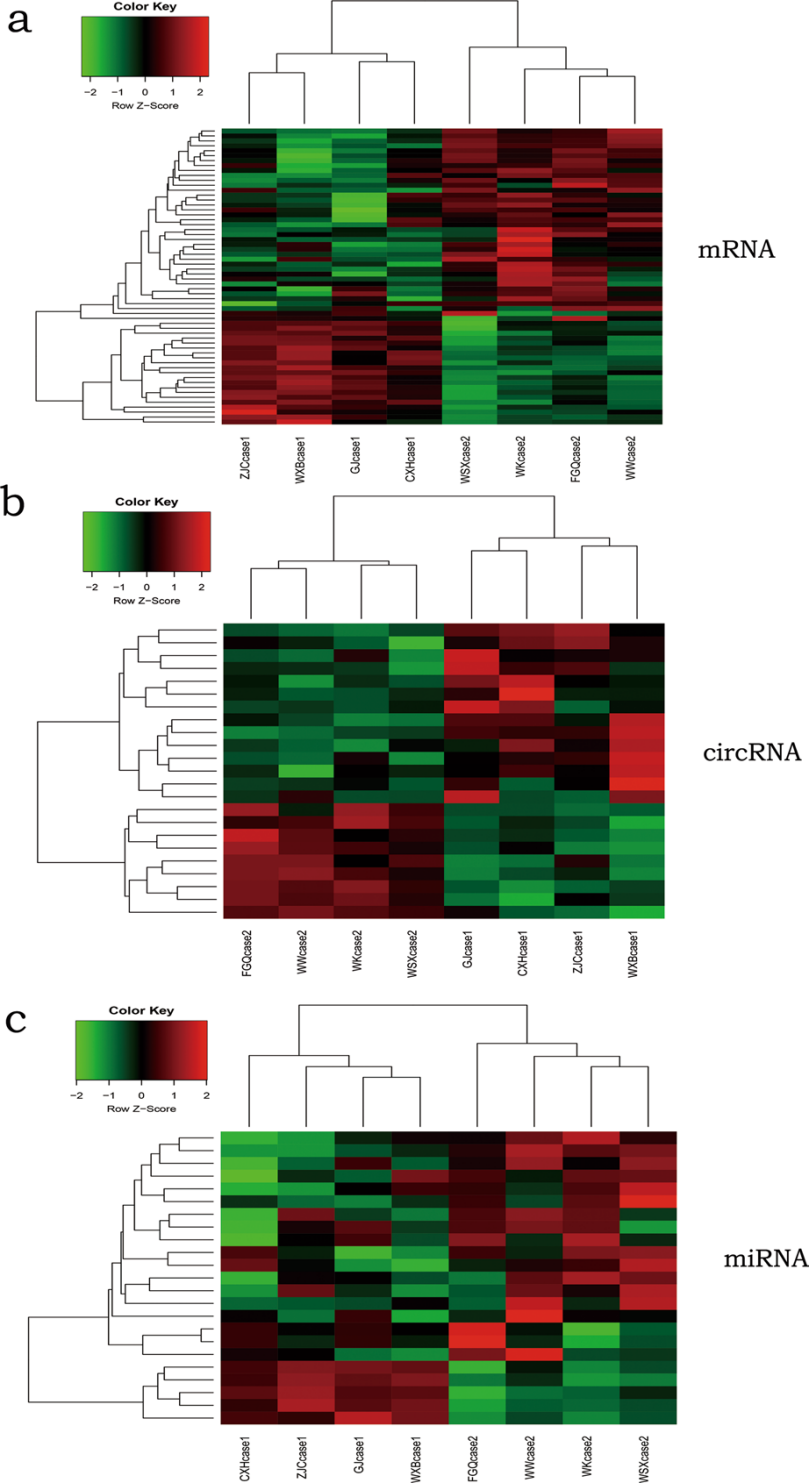
Significance and reliability of differentially expressed RNAs between the two groups. **a** mRNAs; **b** circRNAs; **c** miRNAs

Totally, 15,806 mRNAs, 5088 miRNAs, 20,370 lncRNAs and 57,063 circRNAs were discovered from the cohort of patients from group A, and the corresponding number were 30,293, 5013, 20,685 and 60,205 from the other cohort of patients from group B, respectively. Comparing with group A, 556 mRNAs, 120 circRNAs, 87 lncRNAs, and 96 miRNAs were differentially expressed in group B. Finally, we identified top 8 of mRNAs, circRNAs and lncRNAs that associate HCC patients who IFN-α therapy as showed in heat map (Fig. 1a, b, c).

### The ceRNA network

**Fig. 2:**
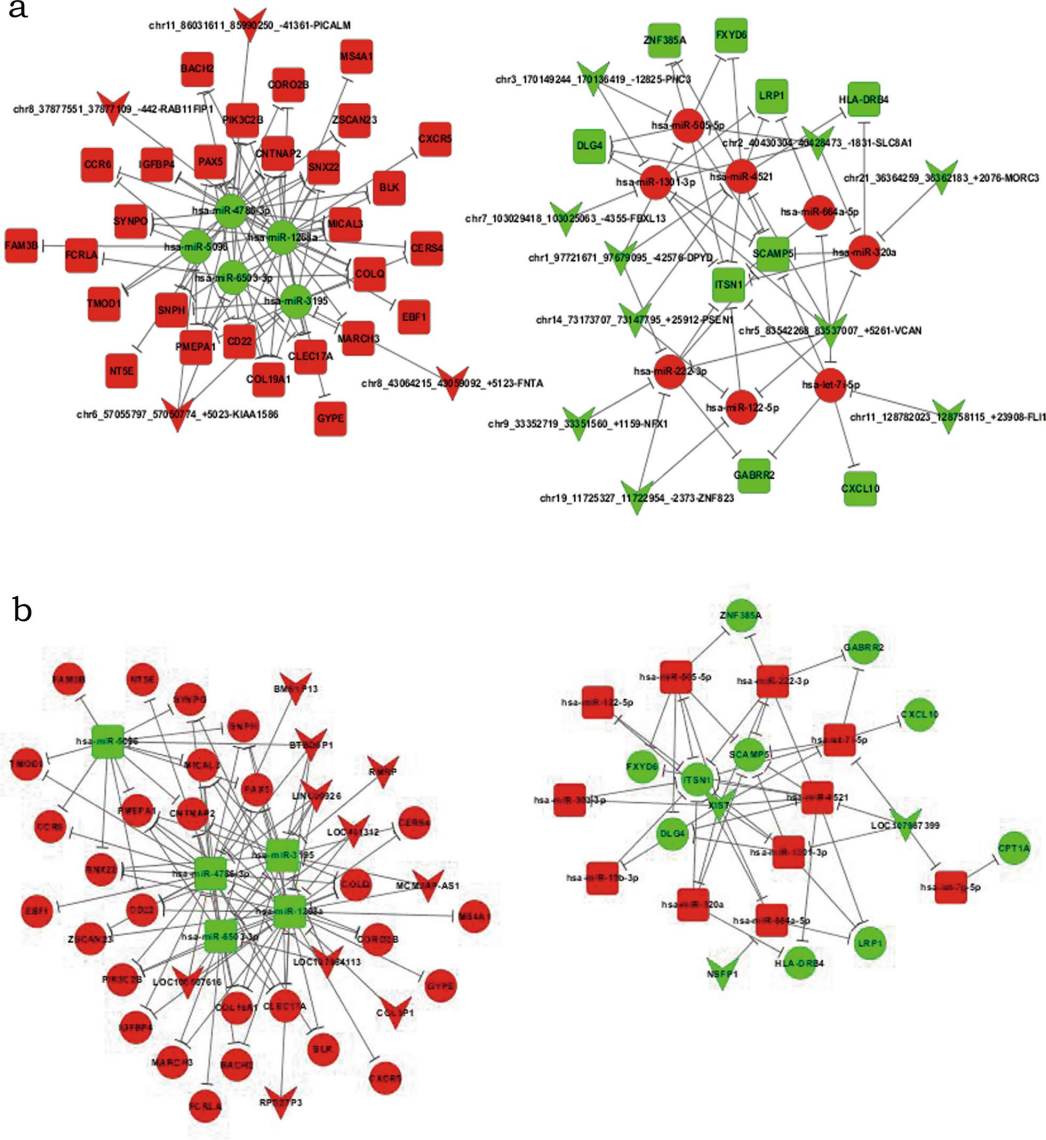
The ceRNA regulatory network of differentially expressed mRNAs under the IFN-α therapy. **a** The circRNA-miRNA-mRNA network. **b** The lncRNA-miRNA-mRNA network. Red, up-regulated; Green, down-regulated

The ceRNA regulatory network was described as follows. In the up-regulated circRNA-miRNA-mRNA network, there were 4 circRNAs, 28 mRNAs and 5 miRNAs, and in the corresponding down-regulated network, 10 circRNAs, 9 mRNAs, and 8 miRNAs were involved (Fig. 2a). The up-regulated lncRNA-miRNA-mRNA network included 10 lncRNAs, 28 mRNAs and 5 miRNAs, and the down-regulated network contained 3 lncRNAs, 10 mRNAs and 11miRNAs (Fig. 2b).

### Functional annotation of the ceRNA network

**Fig. 3:**
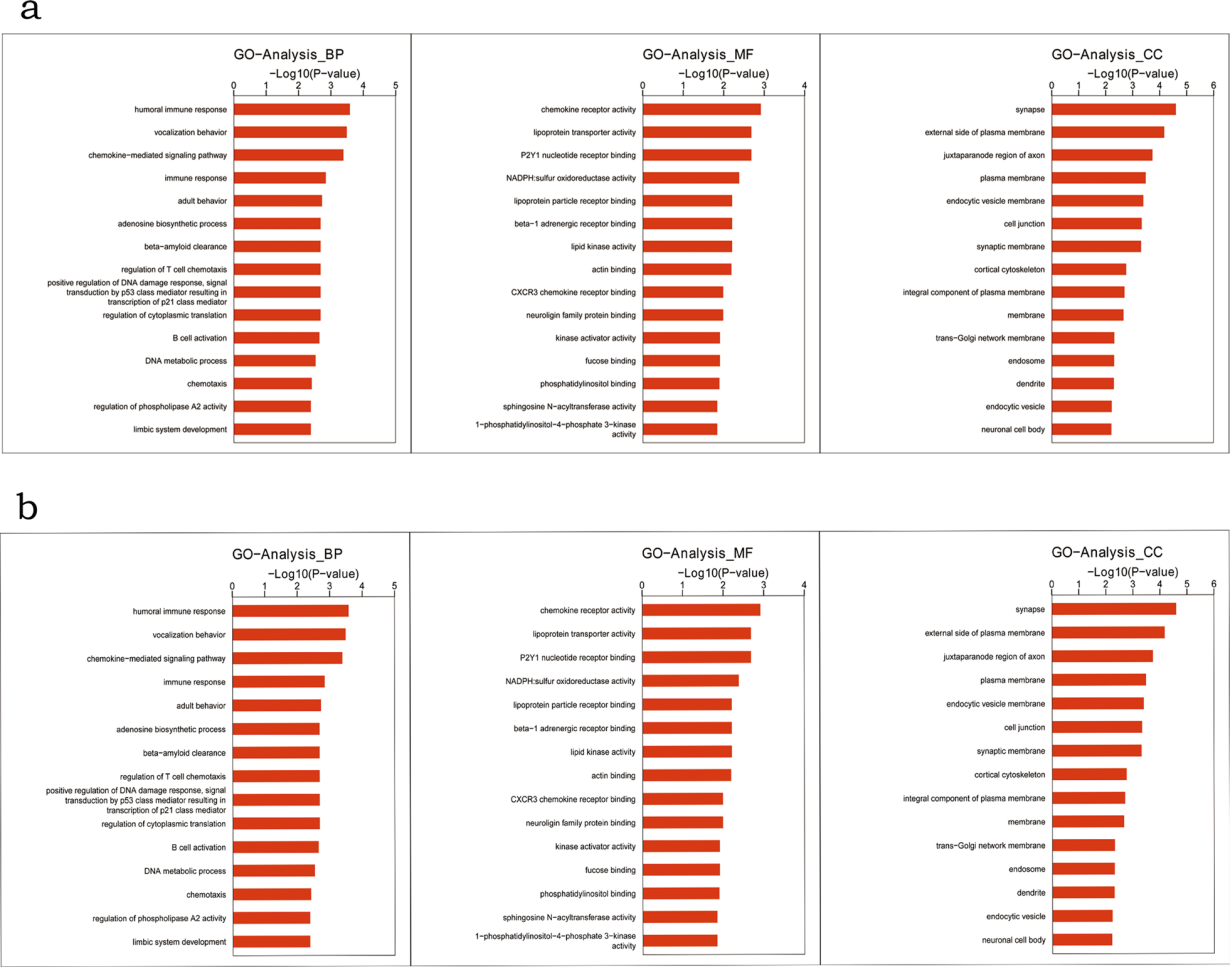
Gene oncology analysis for differentially expressed RNAs involved in the ceRNA network. **a** Genes in the circRNA-miRNA-mRNA network. **b** Genes in the lncRNA-miRNA-mRNA network. BP, biological process; MF, molecular function; CC, cellular component

The GO and KEGG analysis were performed to understand the biological function in the DE-ceRNA network. The result of GO analysis was shown in Fig. 3. Firstly, for circRNA-miRNA-mRNA network, the genes were mainly enriched in synapse, external side of the plasma membrane, and juxtaparanode region of axon; the most enriched GO terms in molecular function (MF) were chemokine receptor activity, lipoprotein transporter activity and P2Y1 nucleotide receptor binding; in additional, humoral immune response, vocalization behavior, chemokine-mediated signaling pathway and immune response were the mainly enriched biological process (BP). For lncRNA-miRNA-mRNA network, the enriched terms of cellular component (CC), MF and BP were basically similar with that of the circRNA-miRNA-mRNA network.

**Fig. 4:**
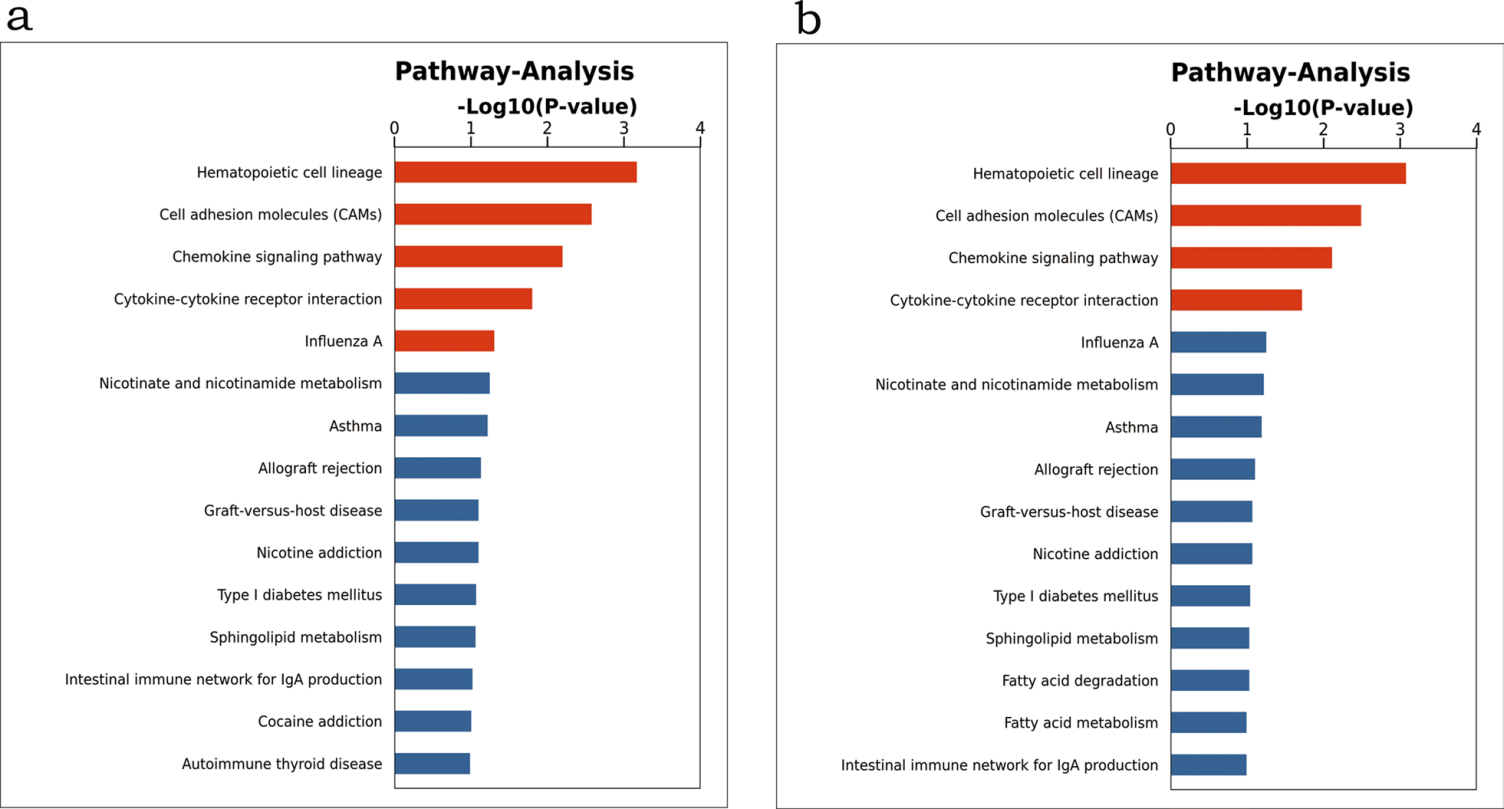
The enriched KEGG pathway of differentially expressed RNAs involved in the ceRNA network under the IFN-α therapy. **a** The up-regulated RNAs networks; **b** The down-regulated RNAs networks. KEGG, Kyoto Encyclopedia of Genes and Genomes; Red, positively regulated pathways; Blue, negatively regulated pathways

Then, we performed the pathway enrichment analysis of the ceRNA network (Fig. 4a, b). The up-regulated RNAs networks including the circRNA-miRNA-mRNA and lncRNA-miRNA-mRNA were both enriched in the five pathways, namely hematopoietic cell lineage, cell adhesion molecules (CAMs), chemokine signaling pathway, cytokine-cytokine receptor interaction, and influenza A. And the down-regulated RNAs network mainly mapped on the following pathways, including nicotinate and nicotinamide metabolism, asthma, and allograft rejection.

### Survival analysis of pivotal genes

**Fig. 5:**
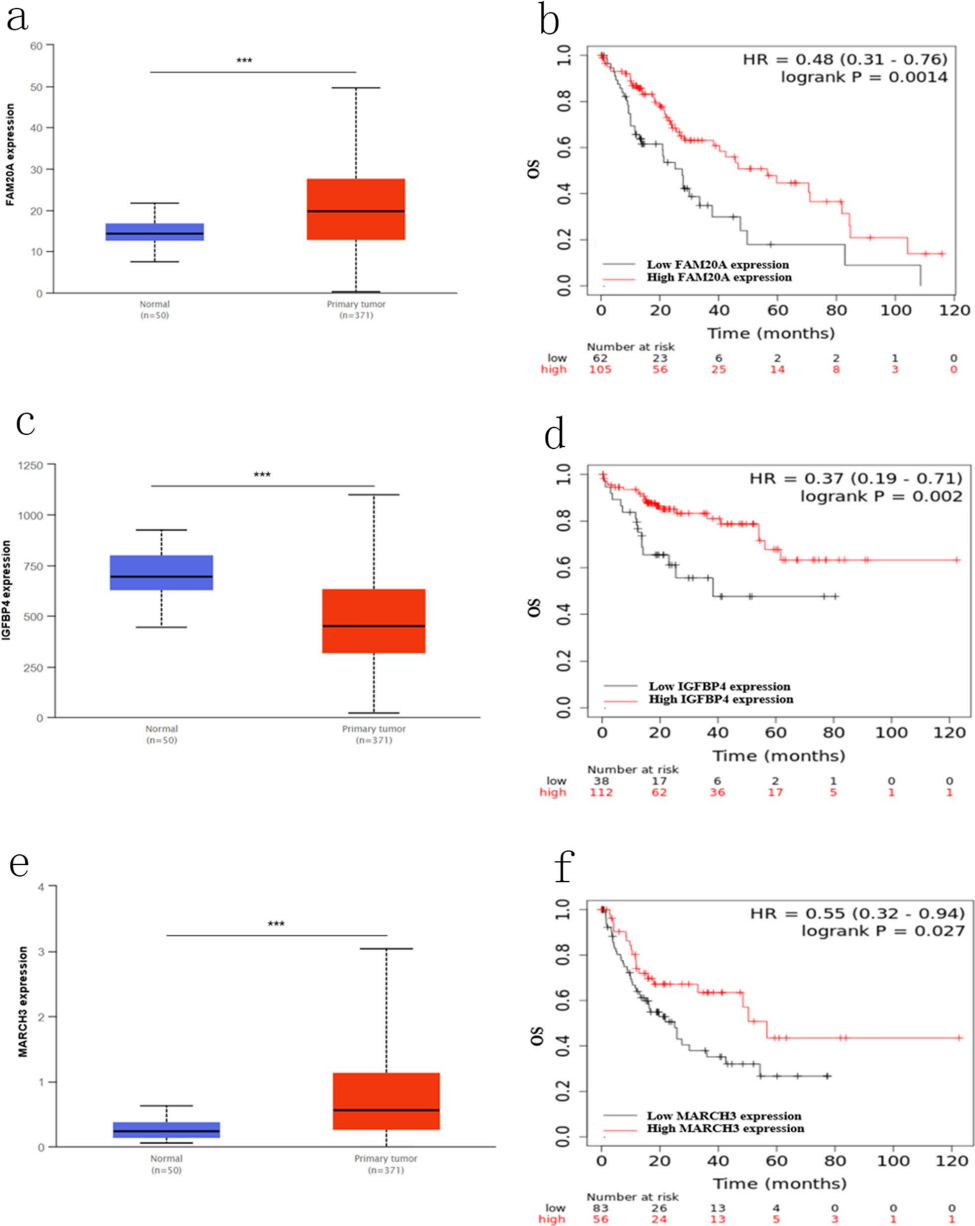
The effect of MARCH3 on immunological status in HCC. **a** Correlation between MARCH3 and 112 immunomodulators (immunoinhibitor, immunostimulator, MHC, receptor, chemokine). **b** Correlation between MARCH3 and three representative immune checkpoints, including PD-1, PD-L1 and CTLA4. **c** Correlation between MARCH3 and 28 tumor-associated immune cells. The color indicates the correlation coefficient. The asterisks indicate a statistically significant p-value calculated using spearman correlation analysis. (*P < 0.05; **P < 0.01; ***P < 0.001)

We utilized the data from TCGA to perform the expression pattern and survival analysis of pivotal genes in HBV related HCC. Only three genes among the DE-ceRNA regulatory network were related with the prognosis, such as FAM20A, IGFBP4 and MARCH3. Notably, FAM20A was upregulated in HCC, and high FAM20A expression correlated with worse OS (p = 0.0014) (Fig. 5a, b). IGFBP4 expression was decreased in HCC, while patients with higher IGFBP4 expression had longer OS (p = 0.002) (Fig. 5c, d). MARCH3 was highly expressed in HCC, and high MARCH3 was associated with shorter OS (p = 0.002) (Fig. 5e, f). These findings suggested FAM20A, IGFBP4 and MARCH3 may be prognostic markers for HBV related HCC. Interestingly, unlike to FAM20A and IGFBP4, MARCH3 was identified as novel biomarker for HCC which has not yet been reported.

### The role of MARCH3 as an immunological regulation in HCC

**Fig. 6:**
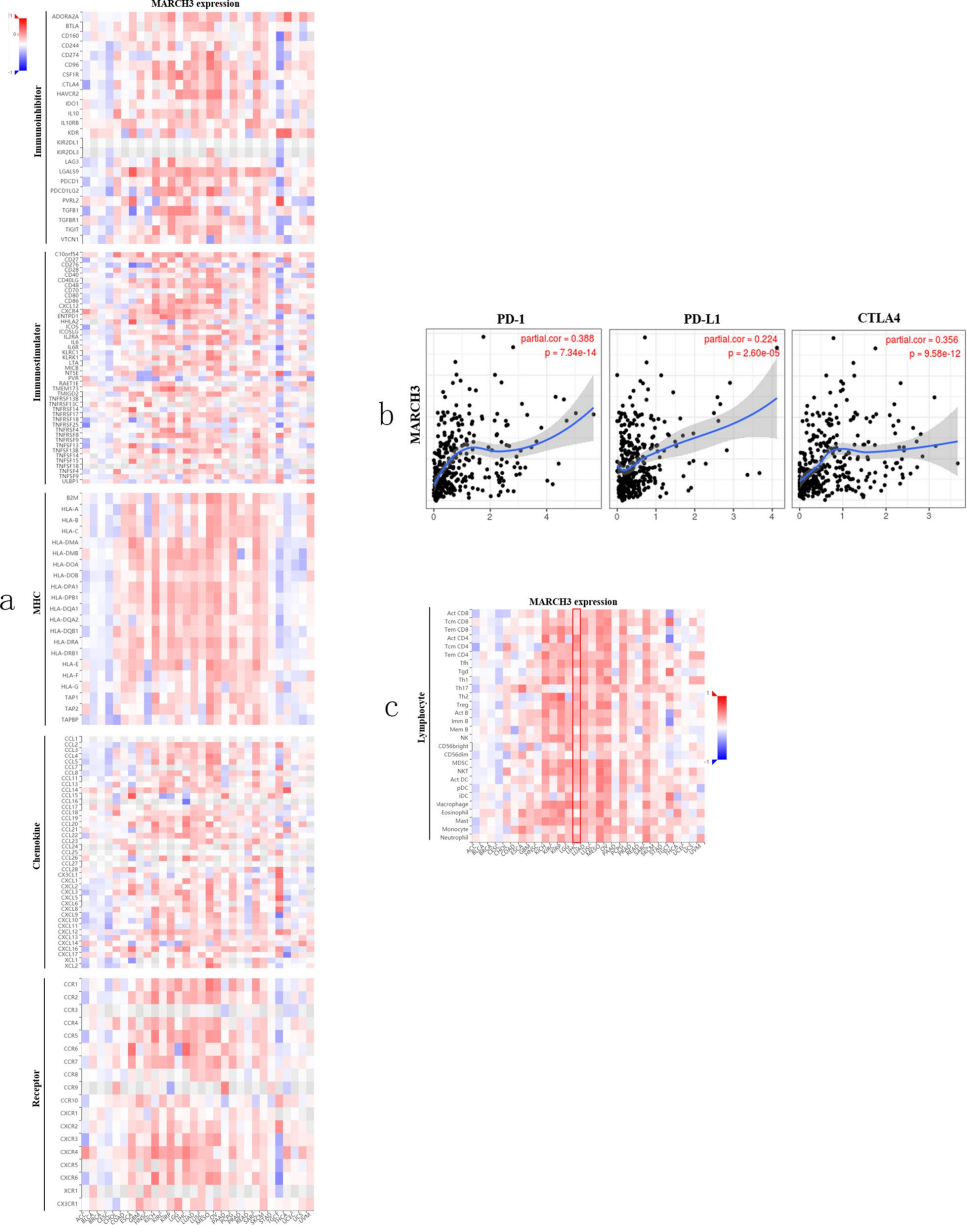
The expression pattern and survival analysis for differentially expressed genes most relevant to overall survival of HCC patients. **a**, **c**, **e** The expression levels of FAM20A, IGFBP4 and MARCH3 in HCC; **b**, **d**, **f** Kaplan–Meier curves for the OS in HCC, stratified by the expression of FAM20A, IGFBP4 and MARCH3. OS, overall survival. P < 0.05 means statically significance

Based on the above findings, we aimed to explore the role of MARCH3 in HCC. Membrane associated ring-CH-type finder 3 (MARCH3), belongs to a membrane-associated RING-CH family, is associated with immune and inflammatory. Analysis of TISIDB database, we aimed to depict the immunological role of MARCH3 are critical in determining the subpopulation of HCC patients that may benefit from target-MARCH3 immunotherapy. Our data showed that MARCH3 was positively associated with a majority of immunomodulators including immunoinhibitor, immunostimulator, major histocompatibility complex (MHC), chemokine and receptor (Fig. 6a). We also estimated the infiltration levels of tumor immune infiltrating cells in the HCC TME. The results revealed that MARCH3 was positively correlated with TIICs in HCC (Fig. 6c). Furthermore, we demonstrated that co-occurrence with immune checkpoints, including PD-1, PD-L1 and CTLA4 in HCC (Fig. 6b). In summary, the expression pattern and immunological status of MARCH3 in HCC is TME specific, which demonstrates the potential of MARCH3 as a target for HCC immunotherapy.

### MARCH3 expression is correlated with immune infiltration in HCC

**Fig. 7:**
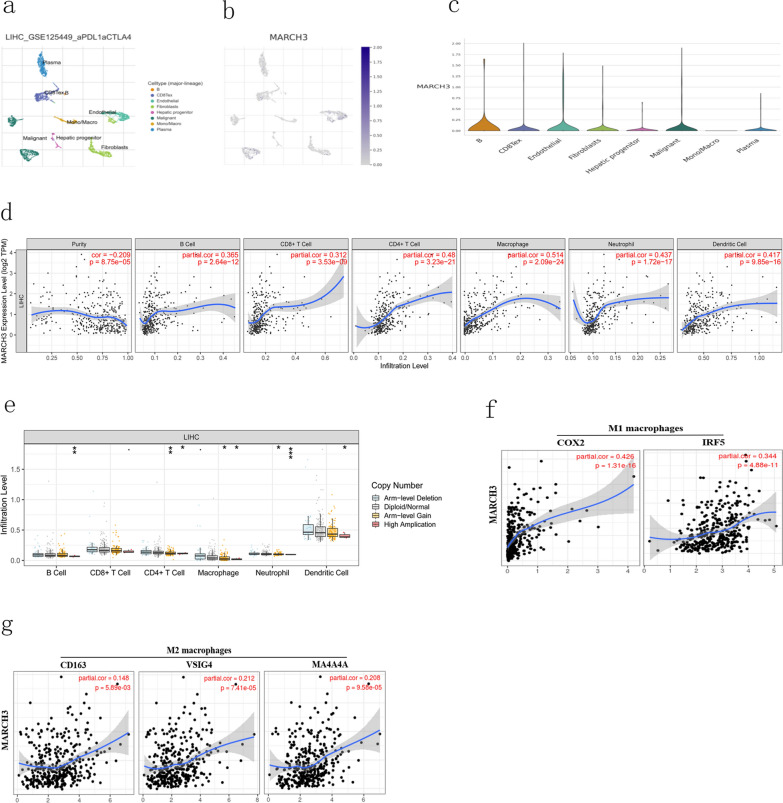
MARCH3 expression correlates with TICCs in HCC. **a** t-SNE projection depicting cellular composition of human HCC tumors. Cells that share similar transcriptome profiles were grouped by colors and were annotated using lineage specific markers. **b**, **c** t-SNE plot depicting MARCH3 in the cells including B cells, CD8 + T cells, endothelial cells, fibroblast cells, hepatic progenitor cells, malignant cells, macrophage cells and plasma cells. **d** Correlation between MARCH3 expression with tumor associated immune cells in HCC. **e** Correlation between MARCH3 alternations (arm-level deletion, diploid/normal, arm level gain and high amplication) and the infiltrating levels of immune cells. **f**, **g** Correlation between MARCH3 expression and the gene markers of M1 macrophages (COX2 and IRF5) and M2 macrophages (cd163, VSIG4 and MA4A4A) in HCC

To further demonstrate the role of MARCH3 as an immune regulatory in HCC, we utilized publicly available single cell RNA seq (scRNA-seq) data from 10 patients, encompassing both malignant and non-malignant cell types and post data processing to investigate the expression level of MARCH3 in TIICs in HCC. We identified 11 clusters annotated to B cells, CD4 + T cells, CD8 + T cells, endothelial cells, fibroblasts cells, hepatic progenitor cells, malignant cells, monocyte/macrophage cells, plasma cells and Tprolif cells (Fig. 7a). MARCH3 was found to be highly expressed in a mount of cells including B cells, CD8 + T cells, endothelial cells, fibroblast cells, hepatic progenitor cells, malignant cells and plasma cells (Fig. 7b, c). Tumor-infiltrating lymphocytes are independent predictors of sentinel lymph node status and survival in cancers. Tumor purity is a critical factor that influences the analysis of immune infiltration in clinical samples by genomic approaches. By analyzed the data from TIMER, we found that MARCH3 expression had a negative correlation with tumor purity in CHOL. In line to the results of TISID analysis, MARCH3 was found to had a strong correlation with B cell (r = 0.365, p = 2.64e−12), CD8 + T cell (r = 0.312, p = 3.53e−9), CD4 + T cell (r = 0.417, p = 3.23e−21), macrophage (r = 0.514, p = 2.09e−24), neutrophil (r = 0.437, p = 1.72e−72) and dendritic cell (r = 0.44, p = 9.85e−16) (Fig. 7d). We next accessed how MARCH3 alternation could influence the infiltrating levels of TIICs in HCC. The results showed that the copy number variation (CNV) pattern of MARCH3 including arm-level deletion, diploid/normal, arm-level gain and high amplication was correlated with the infiltrating levels of TIICs. We observed that MARCH3 alteration in arm-level gain decreased the infiltrating levels of CD4 + T cells, macrophages and neutrophil cells, while MARCH3 alteration in high amplication could also decrease the infiltrating levels of B cells, CD4 + T cells, neutrophil cells and dendritic cells (Fig. 7e). These findings suggest that epigenetic modifications of the MARCH3 gene may be an alternative therapeutic method of intervention for anti-MARCH3 inhibitors. In the above finding, we noticed that MARCH3 expression had the strong correlation with macrophages (R = 0.514, p = 2.09e−24). We hypothesized that MARCH3 overexpression may regulate the macrophages in HCC. To test this hypothesis, we analyzed the correlations between MARCH3 expression and the gene markers of macrophages in HCC. Our data revealed that the gene markers of M1 macrophages such as COX2 (R = 0.426, p = 1.31e−16) and IRF5 (R = 0.344, p = 4.88e−11) showed strong correlations with MARCH3 expression (Fig. 7f), whereas M2 macrophage markers such as CD163 (R = 0.148, p = 5.89e−03), VSIG4 (R = 0.212, p = 7.41e−05) and MS4A4A (R = 0.208, p = 9.58e−05) showed weak correlations (Fig. 7g). Therefore, we considered that MARCH3 could inhibit the polarization of macrophages into tumor-associated macrophages.

### Correlation analysis between MARCH3 expression and immune marker sets

TIMER and GEPIA have most of the homologous data from TCGA. Therefore, we used the data from GEPIA database to verity the effects of MARCH3 on TICCs which was analyzed by TIMER database. We investigated the correlations between MARCH3 expression and the immune marker sets of TICCs including CD8 + T cells, T cells (general), B cells, monocytes, TAMs, M1 and M2 macrophages, neutrophils, NK cells and DCs, and the different functional T cells included Th1 cells, Th2 cells, Tfh cells, Th17 cells, Tregs and exhausted T cells in HCC. After the correlation adjustment by purity, the results showed that MARCH3 expression was significantly correlated with most immune marker sets of various immune cells and different T cells in HCC, whose results were consistent with TIMER analysis (Table 2). Therefore, these findings further demonstrated that MARCH3 could regulate immune function to affect the development and progression of HCC.

**Table 2:** Correlation analysis between MARCH3 and related genes and markers of immune cells in HCC by GEPIA analysis

Description	Gene markers	MARCH3	
		Correlation	P-value
CD8 + T cell	CD8A	0.31	9.3e−10
	CD8B	0.25	7.2e−07
T cell	CD3D	0.38	5.2e−14
	CD3E	0.42	1.9e−17
	CD2	0.42	2.1e−17
B cell	CD19	0.32	3.4e−10
	CD79A	0.39	4.1e−15
Monocyte	CD86	0.46	4.4e−21
	CSF1R	0.38	2e−14
TAM	CCL2	0.16	0.0015
	CD68	0.24	2.4e−06
	IL10	0.34	1.7e−11
M1 Macrophage	NOS2	0.13	0.012
	IRF5	0.33	4e−11
	PTGS2	0.47	5.5e−22
M2 Macrophage	CD163	0.16	0.0017
	VSIG4	0.22	1.3e−05
	MS4A4A	0.26	3.4e−07
Neutrophils	CEACAM8	0.13	0.0099
	ITGAM	0.34	2.1e−11
	CCR7	0.41	2e−16
Natural killer cell	KIR2DL1	0.022	0.67
	KIR2DL3	0.12	0.023
	KIR2DL4	0.14	0.0083
	KIR3DL1	-0.093	0.075
	KIR3DL2	0.22	2.9e−05
	KIR3DL3	0.15	0.004
	KIR2DS4	0.0033	0.95
Dendritic cell	HLA-DPB1	0.38	2.2e−14
	HLA-DQB1	0.20	1e−04
	HLA-DRA	0.34	1.1e−11
	HLA-DPA1	0.35	3.3e−12
	BDCA-1(CD1C)	0.45	1.5e−19
	BDCA-4(NRP1)	0.27	1.8e−07
	CD11c (ITGAX)	0.45	6.2e−20
Th1	T-bet (TBX21)	0.21	3.6e−05
	STAT4	0.45	3.9e−20
	STAT1	0.41	1.2e−16
	IFN-γ (IFNG)	0.18	0.00036
	TNF-α (TNF)	0.39	7.1e−15
Th2	GATA3	0.46	1.1e−20
	STAT6	0.14	0.0062
	STAT5A	0.34	2.7e−11
	IL13	0.17	0.00077
Tfh	BCL6	0.067	0.2
	IL21	0.14	0.0055
Th17	STAT3	0.3	3.5e−09
	IL17A	0.093	0.074
Treg	FOXP3	0.12	0.018
	CCR8	0.46	1.6e−20
	STAT5B	0.15	0.0052
	TGFβ (TGFB1)	0.57	4.7e−33
T cell exhaustion	PD-1 (PDCD1)	0.43	2.3e−18
	CTLA4	0.42	8.6e−17
	LAG3	0.18	0.00056
	TIM-3 (HAVCR2)	0.49	1.8e−23
	GZMB	0.068	0.19

^*^P < 0.01, **P < 0.001, ***P < 0.0001. The result of P < 0.05 means statistical significance

## Discussion

In this study, to clarify the regulatory mechanism of IFN-α therapy on HCC, we analyzed the RNA expression profile by RNA sequencing. According to comparing RNA profile of patients with IFN-α or not, totally 556 mRNAs, 120 circRNAs, 87 lncRNAs, and 96 miRNAs were differentially expressed. Then a ceRNA network included the circRNA-miRNA-mRNA and lncRNA-miRNA-mRNA were constructed. Besides, the biological and functional attributes of these significantly differentially expressed RNAs were analyzed by GO and KEGG databases. Immune-related biological progresses were significantly more enriched. Survival analysis showed that three potential prognostic associated genes, namely FAM20A, IGFBP4 and MARCH3. Finally, we further analyzed the correlation between MARCH3 and immune infiltrate in HCC. To our knowledge, this is the first publication to probe the ceRNA network of IFN-α therapy in HCC and investigated the effect of MARCH3 on regulating immune function in HCC.

Of the genes which were involved in the ceRNA network, GO enrichment analysis indicated that these RNAs were mainly enriched in the synapse, external side of the plasma membrane, and juxtaparanode region of axon. IFN-α, a proinflammatory cytokine, which is commonly produced by natural killer cells and T-lymphocytes [19]. As under the normal conditions, these cells minimally entry into the central nervous system, IFN-α is generally not detected in the brain [20]. But several research have revealed that the high expression level of IFN-α can cause hippocampal development and abnormal cerebellar [21, 22]. The effects of IFN-α on neurons is increasingly drawn attention. McHugh has published a comment that IFN-α can stimulate synapse loss [23]. And based on a mouse model, IFN-α inhibits the dendritic outgrowth which may lead to decreasing the rate of synapse formation [24]. Besides, IFN-α has also been proven to provide the enhanced axon protection [25]. And for localizing in the plasma membrane, this result has been widely accepted as many plasma membrane proteins, like tethering and IFITM3, are identified in the interferon-treated cells [26]. In addition, the biological process of GO term and pathway have mapped the immune-related signaling. As we all know, the IFN-α is a family of cytokine mediators which involves in mediating the cellular immune system [27]. The normal interferon pathways always start with binding ligand to form a ternary complex, and then followed by activating the downstream signaling, like mediating the Janus tyrosine kinases [28]. Then the Toll-like receptors, tissue-destructive cytokines, inflammatory factors, cytokines are regulated to activate the STAT. Subsequently, the function of immune effector genes, like chemokines, phagocytic receptors, antiviral proteins, antigen-presenting molecules, are activated [29]. In current research, we discovered that the significantly differential expression of ceRNA mainly mapped on the immune-related pathway, and this result further proven the importance of the relationship between IFN-α and immune pathway.

Furthermore, 3 genes such as FAM20A, IGFBP4 and MARCH3 among the DE-ceRNA regulatory network related with the prognosis were identified. FAM20A, encoding one of the “family with sequence similarity 20” (FAM20) proteins, function in the secretory signaling to promote protein phosphorylation. In human mesenchymal stem cells, previous paper had proven that this protein presented high expressed level following the IFN-α treatment [30]. Although this protein is originally identified from the hematopoietic cell, FAM20A presents a quite restricted high expression in liver tissue [31]. In addition, based on the data from the Human Protein Atlas (HPA, https://www.proteinatlas.org/ENSG00000108950-FAM20A/pathology/liver+cancer#ihc), FAM20A displayed high expressed level in HCC. And what’ more, FAM20A is a prognostic factor in HCC. However, another largely clinical cohorts are needed to further test the above results. IGFBP4 is a specific insulin-like growths factor (IGF) binding protein which binds to IGF or not to tune the cellular activities, like cell proliferation and migration [32, 33]. In vitro experiment has revealed that the IFN-α can increase the mRNA and protein level of IGFBP4 [34]. And this protein, functioning as a tumor suppressor, participates in driving epigenetic reprogramming of the hepatic carcinogenesis [35]. Although currently there is no research on the relationship between IGFBP4 and prognosis in liver cancer, it has been reported to be associated with prognosis in various cancer types, like lung cancer [36]. Our work first revealed that patients with higher IGFBP4 expression had a longer survival time than that with lower IGFBP4 expression. Membrane-associated RING-CH-3 (MARCH3) is one of the membrane-associated MARCH family members which functions as a negative regulator of adaptive immunity [37]. The whole MARCH family includes 11 members, and 9 of them are transmembrane proteins. Many of these family members have been revealed to associate with prognosis. By researching on a mouse model, targeting MARCH1 exhibited significant inhibition of the growth of HCC [38], and MARCH8 has also played a crucial role in NSCLC against carcinogenesis and progression by validating in clinical samples [39]. In this work, although MARCH3 was down-regulated in HCC, we observed patient-specific heterogeneity, with some patients exhibiting MARCH3 downregulation and others upregulation. Regardless of this heterogeneity, which might be explained by tumor cell biodiversity, here we found that high MARCH3 expression in human HCC correlated with better OS. The role of MARCH3 as ab immune regulator is currently under intense investigation. However, its impact on HCC is almost unknown. Whether a molecule can be a target for normalization cancer immunotherapy depends on two important features: TME-specific overexpression and immunosuppressive function. We first explored the immunological status of MARCH3 in HCC, and we observed that MARCH3 expression was positively associated with many of immunomodulators including immunoinhibitor, immunostimulator, major histocompatibility complex (MHC), chemokine and receptor. Also, MARCH3 expression was found to be in correlation with immune infiltrating in HCC. Our data indicates that MARCH3 plays a critical role in regulating the HCC TME. Tumor progression is highly dependent on the specific TME, which is preponderant on and impacts immunotherapy efficiency. That’s to say, MARCH3 alteration in expression may induce the evolution of TME in HCC, thereby affecting tumor progression and even the patients’ response to immunotherapy.

Analyses of the publicly available scRNA-seq data suggested that MARCH3 was expressed in B cells, endothelial cells, fibroblast cells, malignant cells and CD8 + T cells but not in macrophages. However, we found that MARCH3 expression was correlated with the infiltrating level of diverse immune cells. Especially for macrophages, the correlation with MARCH3 expression was highest. Therefore, we hypothesized that MARCH3 may interact macrophages or other immune cells to influence the development and progression of HCC. We next accessed whether MARCH3 alternation could lead to the various degree of TIICs in HCC and found the CNV of MARCH3 occurred with arm-level gain could decrease the infiltrating levels of CD4 + T cells, macrophages and neutrophil cells, while MARCH3 alteration in high amplication could also decreased the infiltrating levels of B cells, CD4 + T cells, neutrophil cells and dendritic cells. Next, we observed that MARCH3 expression had strong correlation with the gene markers of M1 macrophages, while its expression had weak correlation with the gene markers of macrophages. These findings suggest that MARCH3 could inhibit the polarization of macrophages into tumor associated macrophages.

Although the current research constructed a ceRNA network in HCC for probing the regulation mechanism of IFN-α for the first time, potential limitations existed. First, merely 8 HCC blood samples were obtained from the clinical patients which might lead to the selection bias. Second, in the current research, we haven’t provided the validation data on the discovered results based on the downstream experiments. Anyway, a clinical cohort in larger patient sizes and a following validated experiment are unmet needed in the future.

**Conclusions**

We constructed a ceRNA network and analyzed the biological function of RNAs with significantly differential expression in HCC on researching the effect from IFN-α. The results indicated that immune-related pathways played crucial role in participating in IFN-α treatment. Three genes (FAM20A, IGFBP4 and MARCH3) were identified as the prognostic markers, respectively. Finally, we identified MARCH3 as a vital factor in recruitment and regulation of immune infiltrating cells in HCC. These results laid the foundation on understanding the regulatory mechanism of IFN-α treatment.

## Data Availability

The data sets generated and analyzed during the current study are available from the corresponding author on reasonable request.

## Abbreviations

HCC: Hepatocellular carcinoma
IFN-α: Interferon-alpha
ceRNAs: Competing endogenous RNAs
lncRNA: Long non-coding RNA
miRNA: MicroRNA
circRNA: Circular RNA
DE: Differential expressed
RIG-I: Retinoic acid-inducible gene-I
DPYD: Dihydropyrimidine dehydrogenase
TICCs: Tumor infiltrating immune cells
AS: Antisense RNA
TCGA: The Cancer Genome Atlas
PBMCs: Peripheral blood samples
GO: Gene ontology
KEGG: Kyoto Encyclopedia of Genes and Genomes
GSEA: Gene set enrichment analysis
HBV: Hepatitis B virus
TISCH: Tumor Immune Single Cell Hub
TME: Tumor microenvironment
t-SNE: T-distributed Stochastic Neighbor Embedding
GTEx: Genotype tissue expression
cBioPortal: CBio Cancer Genomics Portal
MF: Molecular function
BP: Biological process
CC: Cellular component
CAMs: Cell adhesion molecules
MARCH3: Membrane associated ring-CH-type finder 3
FAM20: Family with sequence similarity 20
scRNA-seq: Single cell RNA sequence
CNV: Copy number variation
HPA: Human Protein Atlas
MHC: Major histocompatibility complex
